# Laparoscopic or Open Radical Hysterectomy for Early Stage Cancer Cervix: Data Inconsistency of LACC Trial

**DOI:** 10.31557/APJCP.2019.20.10.2881

**Published:** 2019

**Authors:** Shashank Shekhar, L Jeyaseelan

**Affiliations:** 1 *Obstetrics and Gynecology All India Institute of Medical Sciences, Jodhpur, Rajasthan, *; 2 *Department of Biostatistics, Christian Medial College, Vellore, Tamilnadu, India. *

**Dear Editor**

We are commenting upon trial by Ramirez PT et al published in New England Journal of Medicine (November 2018), which compared minimally invasive radical hysterectomy and open radical hysterectomy for early stage cervical cancer (Ramirez et al., 2018). Minimally invasive radical hysterectomy was associated with lower rates of disease-free survival and overall survival. The results of this phase III randomized trial have churned up a great debate within academic circles. A number of comments have appeared in literature where possible reasons for poor survival in survivors of minimally invasive radical hysterectomy group have been discussed. However, to the best of our knowledge no one has discussed the published and supplementary data and methods of the trial in detail. The strength of any randomized trial is evident when the baseline characteristics of patients are similar across different groups and such was the case in LACC Trial or so does it appear. However, in LACC trial, 58 patients (30 in Open and 28 in MIS) did not undergo any surgery (patients withdrew or surgery aborted). The reasons for their withdrawal have not been listed and whether this was observed across all centers or was is limited to few centers is hitherto unpublished. For the purpose of comparing the baseline characteristic authors have included the data belonging to these missing 58 patients and probably justified it on intention to treat analysis. To us it appears arbitrary and flawed. Out of 282 patients who were left in the open laparotomy group, eight patients underwent Laparoscopic or robotic radical hysterectomy. To count these eight subjects in open laparotomy group based on intention to treat analysis is just and logical. Assigning patients to either group was based on randomization but did the withdrawal of 58 subjects follow a random pattern? If not, then there is a strong possibility that their exit might have led to serious imbalance in the baseline distribution of important prognostic predictors of survival such as stage and grade. However, it may be argued that the effect of exodus of 58 subjects on comparability of two groups can still be assessed by the final post-operative histopathological findings. Unfortunately, however, there are gaps in the post-operative histopathological data aswell. Grade of the tumor is not reported in nearly 30 % subjects in each group. Lymph vascular space invasion is not reported in another one third patients in each group. In final HPR there is no attempt to distinguish between micro invasive stage (and substages) and stage 1B1 as both have been clubbed together under umbrella group of < 2cm tumor size. A Kaplan meier survival analysis inherently adjusts the data lost due to censoring. However, for a good survival analysis it is imperative that that not more than 50% data should be censored (Lee and Wang, 2003). In LACC trial nearly 70% data was censored at 4.5 years as is clear from the Kaplan-meier survival chart and at 5 years almost 98% data was censored. 

We want to congratulate the authors for this mammoth trial spanning 10 year across the globe. It was a herculean task and authors deserve every bit of appreciation. However, the debate this trial has generated must address the question that whether the evidence generated is robust enough to change clinical practices in the light of data inconsistencies we have highlighted. 
